# Prevention of muscle atrophy in ICU patients without nerve injury by neuromuscular electrical stimulation: a randomized controlled study

**DOI:** 10.1186/s12891-022-05739-2

**Published:** 2022-08-16

**Authors:** Weiwei Bao, Jiajia Yang, Mingna Li, Kang Chen, Zheng Ma, Yuehong Bai, Yiming Xu

**Affiliations:** 1 Department of Rehabilitation Medicine, Shanghai Jiao Tong University affiliated Sixth People’s Hospital, Shanghai, China; 2 The First Affiliated Hospital, Sun Yat-sen University, Guangzhou, China; 3 Department of Critical Care Medicine, Shanghai Jiao Tong University affiliated Sixth People’s Hospital, Shanghai, China

**Keywords:** NMES, Muscular atrophy, ICU, Prevention, Strategy, Efficacy, Safety

## Abstract

**Background:**

Extensive muscle atrophy is a common occurrence in orthopaedics patients who are bedridden or immobilized. The incidence is higher in intensive care unit (ICU) inpatients. There is still controversy about how to use neuromuscular electrical stimulation (NMES) in ICU patients. We aim to compare the effectiveness and safety of NMES to prevent muscle atrophy in intensive care unit (ICU) patients without nerve injury.

**Methods:**

ICU patients without central and peripheral nerve injury were randomized into experimental group I (Exp I: active and passive activity training (APAT) + NMES treatment on the gastrocnemius and tibialis anterior muscle), experimental group II (Exp II: APAT + NMES treatment on gastrocnemius alone), and control group (Ctl: APAT alone). Changes in the strength of gastrocnemius, the ankle range of motion, and the muscle cross-section area of the lower leg were evaluated before and after the intervention. Also, changes in prothrombin time, lactic acid, and C-reactive protein were monitored during the treatment.

**Results:**

The gastrocnemius muscle strength, ankle joint range of motion, and cross-sectional muscle area of the lower leg in the three groups showed a downward trend, indicating that the overall trend of muscle atrophy in ICU patients was irreversible. The decrease in gastrocnemius muscle strength in Exp I and Exp II was smaller than that in the control group (*P* < 0.05), but there was no difference between Exp I and Exp II. The decrease in active ankle range of motion and cross-sectional area of the lower leg Exp I and Exp II was smaller than that in the control group (*P* < 0.05), and the decrease in Exp I was smaller than that of Exp II (all *P* < 0.05). The curative effect in Exp I was better than in Exp II. There were no significant differences in the dynamic changes of prothrombin time, lactic acid, and C-reactive protein during the three groups (*P* > 0.05).

**Conclusion:**

In addition to early exercise training, NMES should be applied to prevent muscle atrophy for patients without nerve injury in ICU. Also, simultaneous NMES treatment on agonist/antagonist muscle can enhance the effect of preventing muscle atrophy.

**Trial registration:**

This study was prospectively registered in China Clinical Trial Registry (www.chictr.org.cn) on 16/05/2020 as ChiCTR2000032950.

## Background

Extensive muscle atrophy is a common occurrence in patients who are bedridden or immobilized [1, 2], while the degree of muscle atrophy is positively correlated with the time spent in bed [1]. Also, the incidence is higher in intensive care unit (ICU) inpatients. Disturbance of consciousness, mechanical ventilation, use of glucocorticoids, insufficient nutritional intake, and so on are some of the factors that can reduce muscle protein synthesis and promote muscle protein decomposition in ICU patients. Also, some ICU patients developed intensive care unit-acquired weakness (ICU-AW) [3, 4]. Surveys have shown that the incidence of muscular atrophy in intensive care patients receiving mechanical ventilation is as high as 60% [5, 6]. In addition, muscle atrophy has been closely associated with a prolonged hospital stay, increased duration of mechanical ventilation, and increased mortality [7, 8]. Also, studies have reported that muscle atrophy develops rapidly during the first week of stay in the ICU [1, 9, 10]; thus, early and effective intervention is very important.

Among early interventions, Neuromuscular Electrical Stimulation (NMES) has been commonly used to prevent muscle atrophy in ICU patients by improving their muscle strength and maintaining muscle mass [11, 12]. Yet, whether NMES should be used for conscious ICU bedridden patients who can independently move remains debatable. Some scholars advocate that NMES should not be used when the patient’s consciousness level is improved, and the patient can carry out autonomous activities [13], while other studies suggested that early active contraction combined with NMES can more alleviate muscle strength loss and atrophy through different modes of muscle activation [14]. Moreover, some studies have shown a dose-response relationship between NMES treatment intensity and NMES effectiveness [15], i.e., the non-physiological high stimulation intensity and disordered recruitment of motor units caused by NMES may lead to rapid muscle fatigue and muscle injury [16–18]. On the other hand, when the agonist and antagonist muscles are stimulated, the muscle fibers contract synchronously, reducing fatigue [19]. Still, it remains unclear whether simultaneous stimulation of the agonist and antagonist muscle can better prevent muscle atrophy.

In this study, we explored whether NMES treatment should be added to ICU patients without neurological impairment who can carry out active activities in bed. We compared the efficacy of NMSE treatment when simultaneously stimulating agonist and antagonist muscles. In addition, the safety of early NMES intervention was analyzed and discussed.

## Methods

### Study design

This study was a randomized parallel controlled trial and approved by the Ethics Committee of Shanghai Sixth People’s Hospital (2020–076) and registered in the China Clinical Trial Registry (ChiCTR2000032950) on 16/05/2020. All patients signed the informed consent form. All methods were performed in accordance with the Declaration of Helsinki. Furthermore, the trial was reported based on the Guidelines for Consolidated Standards of Reporting Trials (CONSORT 2010).

### Subjects

Patients admitted to the emergency ICU of our hospital from December 2020 to June 2021 were included in the study. Inclusion criteria were: (1) patients who were conscious; (2) with no central and peripheral nervous system injury; (3) no acute exacerbation; (4) expected to be treated in ICU for more than 1 week, and training could be completed on at least one side of the lower limbs. Exclusion criteria were the following: patients in a coma, inability to cooperate with treatment, original limb function defect or neuromuscular disease, other defects, wound or external fixation of the treatment area, patients with sarcopenia (calf circumference < 34 cm for men and < 33 cm for women, and grip strength < 28 kg for men and < 18 kg for women) [20], and other contraindications of NMES, such as high fever, cardiac pacemaker implantation, severe arrhythmia, etc.

The changes in the muscle strength of the gastrocnemius measured by dynamometer before and after 1 week of bed rest were selected as the main outcome, then based on the mean ± standard deviation (20.69 ± 5.24, 18.45 ± 5.13) the effect value was calculated by GPower3.1(University of Düsseldorf, Germany) as 0.43. The sample size was calculated as 57 cases (α = 0.05, 1-β = 0.8). Therefore, taking into account the 15% loss rate, the proposed sample is 65 cases.

**Fig. 1:**
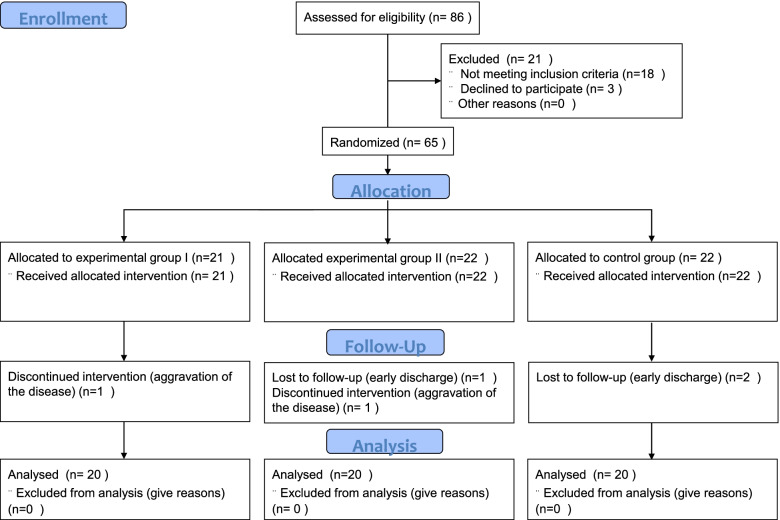
Flow diagram of the study

The patients who met the trial criteria were randomly assigned to three groups using the block randomization method with a block size of eleven: Experimental group I (*n* = 21), Experimental group II (*n* = 22) and Control group (*n* = 22) (Fig. 1). To control the potential selection bias, the random allocation sequence was generated by a person who was not involved in the enrollment or screening of participants. The enrolled patients, the evaluators and the statistical analysis specialist were blinded to group assignment.

### Interventions

Experimental group I (Exp I): patients received active and passive activity training (APAT) on the lower limbs + NMES on the gastrocnemius and tibialis anterior muscles; experimental group II (Exp II): patients received APAT on lower limbs + NMES on gastrocnemius alone; control group (Ctl): patients received APAT on the lower limbs. The initial time of intervention was the 2nd or 3rd day after admission to the ICU ward. The end time of intervention was the day of transfer out of ICU or the day before.

#### Active and passive activity training

Patients were placed in a supine position. The same therapist passively moved the patient’s bilateral ankles, knees, and hips to the maximum range of motion for 5–10 minutes and then instructed the patient to perform active dorsiflexion, plantar flexion, inversion and eversion of ankles, active extension and flexion of knees, active flexion of hips, and abduction and adduction to the maximum range of motion. The training lasted 15 to 20 minutes and was performed twice per day for at least 7 days until the patient was released from the ICU. If one side of the patient’s lower limb needed to be immobilized due to illness, only the healthy side of the lower limb was trained.

#### NMES treatment

**Fig. 2:**
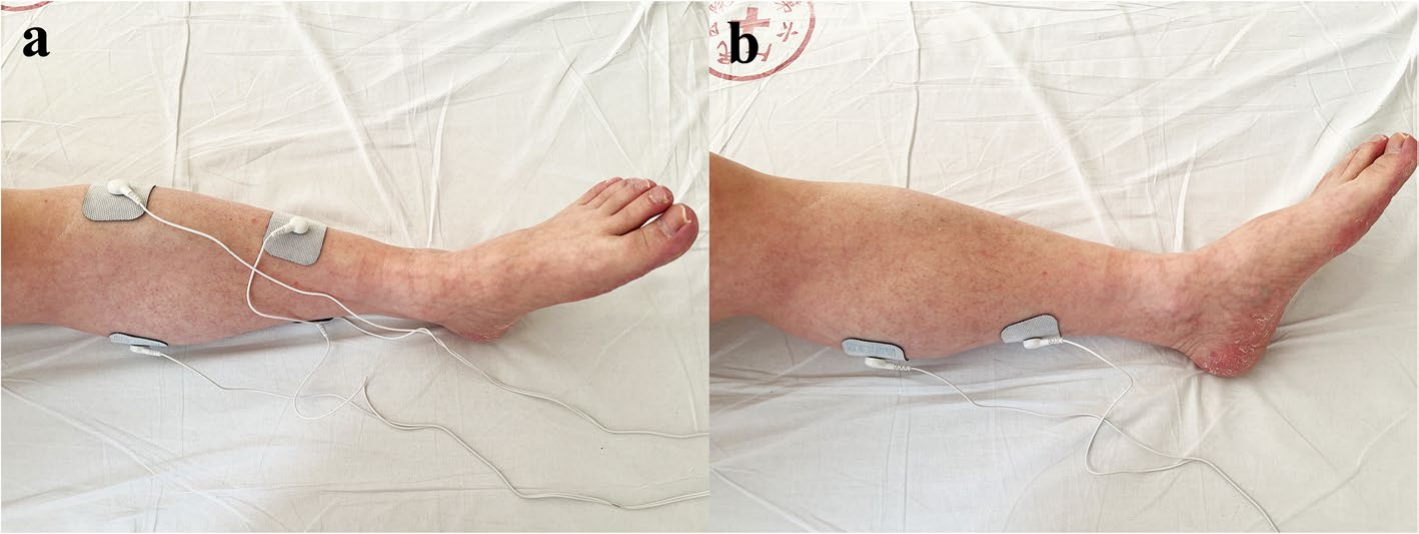
Neuromuscular electrical stimulation treatment. **a** NMES on the gastrocnemius and tibialis anterior muscles in experimental group I . **b** NMES on gastrocnemius alone in experimental group II

Patients were placed in a supine position. The patient’s calfskin was exposed, and then two self-adhesive electrodes (6 cm × 9 cm) were placed on the motor point of the target muscle (the skin area with the lowest stimulation threshold when stimulating the muscle)(Fig. 2). The adhesive area was marked with a marking pen to allow stimulation in the same position every day. The electrode plate was connected with a neuromuscular electrical stimulator (QT-22 T, ITO, Japan). Based on the recommendations of the physiotherapy techniques textbook, for mild to moderate disuse myatrophy, the parameters are set as follows: pulsed current and a biphasic, asymmetrical, balanced rectangular waveform [21, 22], frequency of 30 Hz(the frequency that feels comfortable to the human body [23]), wave width of 300 μs(consistent with the time value for stimulating the motor nerve [24]), on/off ratio of 1:4, adjusting the current intensity according to the patient’s feelings, 20 min/time, twice a day for at least 7 days until the patient was released from the ICU. Effective muscle stimulation is defined as palpable and visible muscle contraction. The same therapist performed the treatment.

### Primary outcome measures

#### Muscle strength of the gastrocnemius

**Fig. 3:**
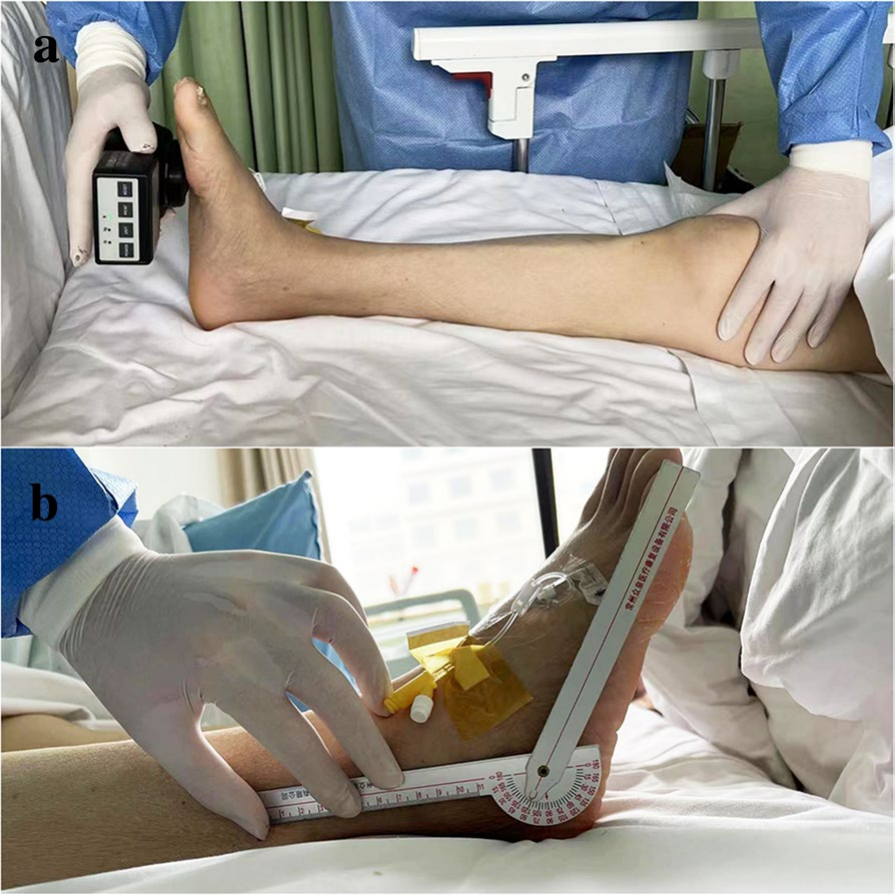
Measure the strength of gastrocnemius muscle (**a**) and Active joint range of motion of ankle joint (**b**)

The muscle strength of ankle plantar flexion (gastrocnemius) was measured with a dynamometer (OE-210, ITO, Japan) at baseline and at the same time (8: 00 ~ 9: 00 am) before transferring the patient out of the ICU. The patient was placed in the supine position; the hip and knee joints were in the extension position, and the ankle joints were in the neutral position. The therapist fixed the knee joints with one hand and placed the dynamometer on the proximal end of the metatarsal bone in the sole resisting the plantarflexion force with the other hand. Patients were instructed to perform plantar flexion of the ankle to maximum isometric contraction during the test (Fig. 3a). A one-minute rest was given between two consecutive tests, and the measurements were repeated 3 times; data were averaged. Only the lower extremities that received the therapeutic intervention were assessed, and for patients who received the intervention in both lower extremities, the results were averaged and recorded. The assessment was completed by the same therapist who was blinded to the grouping.

#### CT evaluation of muscle cross-sectional area

**Fig. 4:**
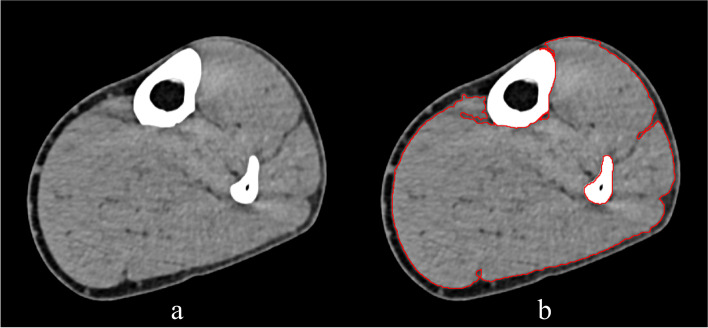
Lower leg CT plain scan and muscle area marking. **a** CT cross-section image of the lower leg (10 cm below the tibial tubercle). **b** Muscle boundary was marked with ImageJ; the cross-sectional area of the muscle was automatically calculated

Patients underwent CT plain scan on the lower leg of the treatment side when entering the ICU and then again before leaving the ICU. Before scanning, the non-metallic marker was placed at 10 cm below the tibial tubercle. CT images of the lower leg were obtained by the plain scan with CT (SOMATOM Force, SIEMENS, Germany). The muscle boundary of the target section was semi-automatically marked by ImageJ (Fig. 4) [25], after which the muscle cross-sectional area (CSA) was automatically calculated. After obtaining the data before and after treatment, the value before treatment was defined as 100%, and then the change rate of the value after treatment was calculated. CT scanning, labeling, and recording of CSA data were performed and averaged bilaterally by the same radiology operator who was blinded to grouping.

#### Active joint range of motion of ankle joint

The active range of motion (AROM) of the ankle was measured at baseline and then again before the patient was released from the ICU. The patient was placed in the supine position. The knee joint was straight or slightly flexed, the ankle was in the neutral position; the intersection point of the fibula longitudinal axis and the outer edge of the foot was the axis, the line parallel to the fibula longitudinal axis was the fixed arm, and the line parallel to the fifth metatarsal longitudinal axis was the moving arm. Then ankle active dorsiflexion and plantarflexion angles were measured by goniometer (IL-1, Changzhou Zhongquan Medical Rehabilitation Equipment Co., Ltd., China) before and after treatment (Fig. 3b). AROM = plantar flexion angle + dorsiflexion angle. Each patient was measured twice, and data were averaged. The same therapist who performed the muscle strength assessment completed the measurements.

### Secondary outcome measures

#### Safety of NMES treatment

The changes in C-reactive protein (CRP), prothrombin time (PT), and lactic acid were monitored on days 1, 3, 5, and 7 during the treatment using an automatic CRP analyzer (PA-990, Lifotronic Shenzhen Pumen Technology Co., Ltd., China), automatic coagulation analyzer (CS5100, Sysmex Co., Ltd., Japan), and automatic blood gas analyzer (GEM Premier 3500, Instrumentation Laboratory, USA). The operator of biochemical testing was blinded to the grouping.

### Statistical analysis

SPSS 23.0 (SPSS, IBM, USA) was used to complete data entry and statistical analysis. Enumeration data were expressed as frequency, and measurement data were expressed as mean ± standard deviation ($\overline{\mathrm{x}}$ ± SD). The chi-square test was used to compare enumeration data. When the measurement data were in accordance with the normality test and homogeneity of variance test, the F test was used, on the contrary, the rank-sum test was used. The comparison among the three groups was conducted by repeated measurement data analysis of variance, and the LSD method was used to compare between groups (whether the intervention was implemented or not) and within groups (different time points). A two-sided test was applied, and *P-*value < 0.05 was considered statistically significant.

## Results

### General information

A total of 65 patients were recruited; 3 patients were lost due to early discharge (Exp II 1 case and Ctl 2 cases), and 2 patients were lost due to aggravation of the disease (1 case both in Exp I and Exp II). Finally, 60 patients participated in the study, all of whom belonged to the Per Protocol Set (PPS), including 51 males and 9 females. No adverse events were reported. Most of the patients had pelvic and spinal fractures (72.8%), and the average ICU stay was 13.35 days. The general information about the patients is shown in Table 1. There was no significant difference in the general information among the three groups (all *P* > 0.05).

**Table 1:** General data sheet

	Exp I (*n* = 20)	Exp II (*n* = 20)	Ctl (*n* = 20)	*P*-value
Age($\overline{\mathrm{x}}$±SD)	52.80 ± 10.79	51.10 ± 17.61	52.50 ± 12.51	0.909
Gender (female/male)	6/14	2/18	1/19	0.069
Smoking history (n,%)	3(5)	6(10)	4(6.7)	0.507
Drinking history (n,%)	2(3.3)	3(5)	5(8.3)	0.436
Hypertension (n,%)	2(3.3)	2(3.3)	3(5)	0.207
History of diabetes (n,%)	2(3.3)	1(1.7)	3(5)	0.561
History of coronary disease (n,%)	2(3.3)	0(0)	1(1.7)	0.237
Length of stay ($\overline{\mathrm{x}}$±SD)	14.10 ± 6.05	12.85 ± 4.67	12.20 ± 4.18	0.483
APACHE II($\overline{\mathrm{x}}$±SD)	9.35 ± 4.53	9.65 ± 5.24	9.20 ± 2.98	0.946
Days in bed from injury to pre-intervention($\overline{\mathrm{x}}$±SD)	3.18 ± 0.75	2.92 ± 1.25	3.54 ± 1.32	0.231
Days of intervention($\overline{\mathrm{x}}$±SD)	12.58 ± 4.27	11.41 ± 2.23	11.19 ± 3.28	0.379
Mechanical ventilation (n,%)	4(6.7)	3(5)	3(5)	0.265
Days under mechanical ventilation($\overline{\mathrm{x}}$±SD)	9.00 ± 1.41	10.67 ± 1.53	8.67 ± 1.53	0.711
Operation (n,%)	18(30)	14(23.3)	19(31.7)	0.069
Blood transfusion (n,%)	9(15)	10(16.7)	7(11.7)	0.619
Mechanical ventilation (n,%)	6(10)	6(10)	3(5)	0.427
Anticoagulant use (n,%)	15(25)	16(26.7)	14(23.3)	0.765
Corticosteroid therapy (n,%)	5(8.3)	4(6.7)	8(13.3)	0.350
Use sedatives (n,%)	8(13.3)	6(10)	7(11.7)	0.803
Days of sedative use($\overline{\mathrm{x}}$±SD)	8.75 ± 2.36	8.17 ± 2.04	8.71 ± 12.43	0.879
Days without sedatives^a^($\overline{\mathrm{x}}$±SD)	4.25 ± 1.67	3.67 ± 1.63	3.29 ± 1.11	0.468
Type of diseases				
Multiple injuries (n,%)	16(26.7)	18(30)	20(33.3)	0.108
Pelvic fracture	9(15)	7(11.7)	16(26.7)	
Thoracolumbar fracture	7(11.7)	6(10)	1(1.7)	
Multiple rib fractures	3(5)	5(8.3)	10(16.7)	
Hemorrhagic shock	2(3.3)	2(3.3)	0(0)	
Lung infection	2(3.3)	3(5)	3(5)	
Others (n,%)	4(6.7)	2(3.3)	0(0)	
Bilateral lower limbs were intervened (n,%)	16 (26.7)	17 (28.3)	13 (21.7)	0.298
Unilateral lower limb was intervened (n,%)	4 (6.7)	3 (5)	7 (11.7)	
The number of NMES sessions($\overline{\mathrm{x}}$±SD)	22.55 ± 3.79	21.65 ± 2.78	21.15 ± 3.07	0.389

^a^Days without sedatives = Length of stay - Days of sedative use

For ICU patients, the severity of critical illness, duration of ICU stay, the duration of mechanical ventilation, the duration of sedation and corticosteroid therapy were risk factors for ICU-acquired muscle weakness [3]. In this study, the proportion of patients with those risk factors was not high, and there was no statistical difference among the three groups.

### Primary outcome measures

#### Comparison of gastrocnemius muscle strength

**Fig. 5:**
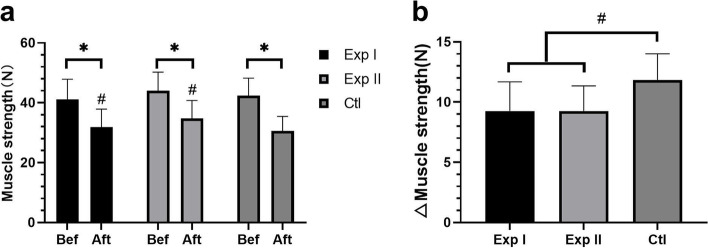
Comparison of gastrocnemius muscle strength before and after treatment. **a** Decrease of gastrocnemius muscle strength before and after treatment in the three groups. **b** Gastrocnemius muscle strength gap before and after treatment among the three groups. Exp I: experimental group I, Exp II: experimental group II, Ctl: control group; Bef: before treatment, Aft: after treatment; * *P* < 0.05 between each group before and after treatment; ^#^ *P* < 0.05 vs. control group

Compared with before treatment, the gastrocnemius muscle strength of the three groups decreased to different degrees (all *P* < 0.05). The muscle strength of Exp I and Exp II was higher than that of the Ctl (Exp I vs Ctl, d = 2.60, 95%CI:1.13 ~ 4.07, *P* < 0.05; Exp II vs Ctl, d = 2.60, 95%CI:1.24 ~ 3.96, *P* < 0.05), but there was no significant difference between Exp I and Exp II (d = 0.00, 95%CI:-1.45 ~ 1.45, *P* > 0.05) (Fig. 5).

#### Comparison of ankle AROM

**Fig. 6:**
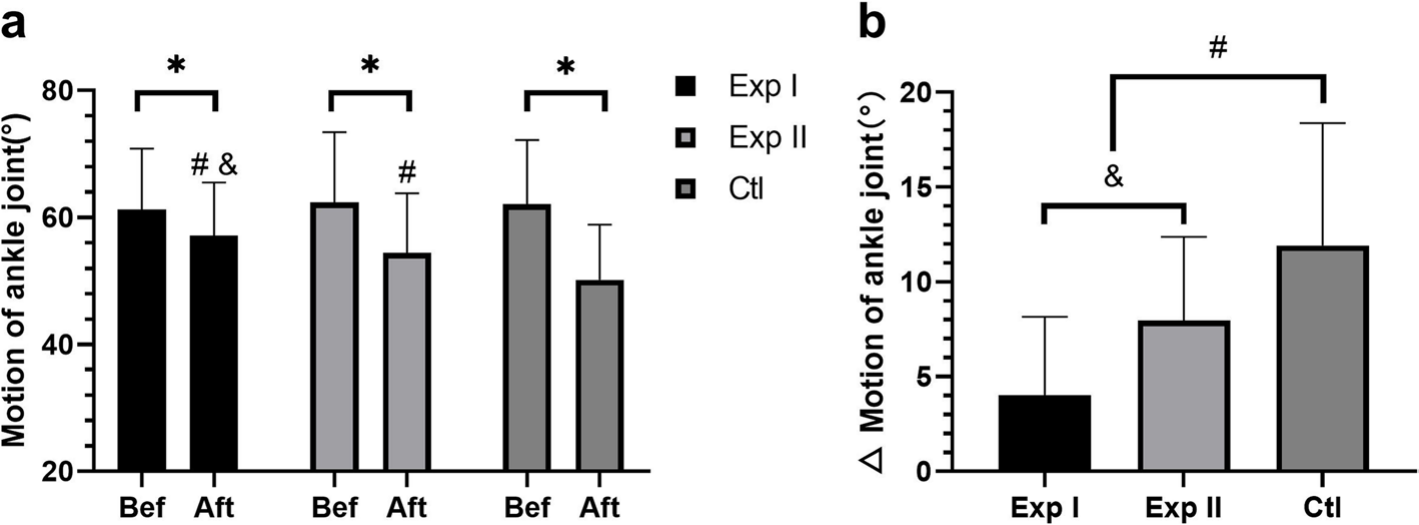
Comparison of AROM of ankle joint before and after treatment. **a** AROM of ankle joint before and after treatment among three groups. **b** Comparison of ankle AROM gap before and after treatment in three groups. Exp I: experimental group I, Exp II: experimental group II, Ctl: control group; Bef: before treatment, Aft: after treatment; * *P* < 0.05 between each group before and after treatment; ^#^ *P* < 0.05 vs. control group; ^&^ *P* < 0.05 between the Exp I and the Exp II

Compared with before treatment, the AROM of ankle joints in three groups decreased to different degrees (all *P* < 0.05). The gap analysis between groups showed that after treatment, the decrease of AROM of the ankle joint in Exp I and Exp II was smaller than that in Ctl(Exp I vs Ctl, d = 7.90, 95%CI:4.437 ~ 11.36, *P* < 0.05; Exp II vs Ctl, d = 3.97, 95%CI: 0.43 ~ 7.50, *P* < 0.01), and there was a significant difference between Exp I and Exp II (d = 3.93, 95%CI: 1.19 ~ 6.67, *P* < 0.05) (Fig. 6).

#### Comparison of muscle CSA

**Fig. 7:**
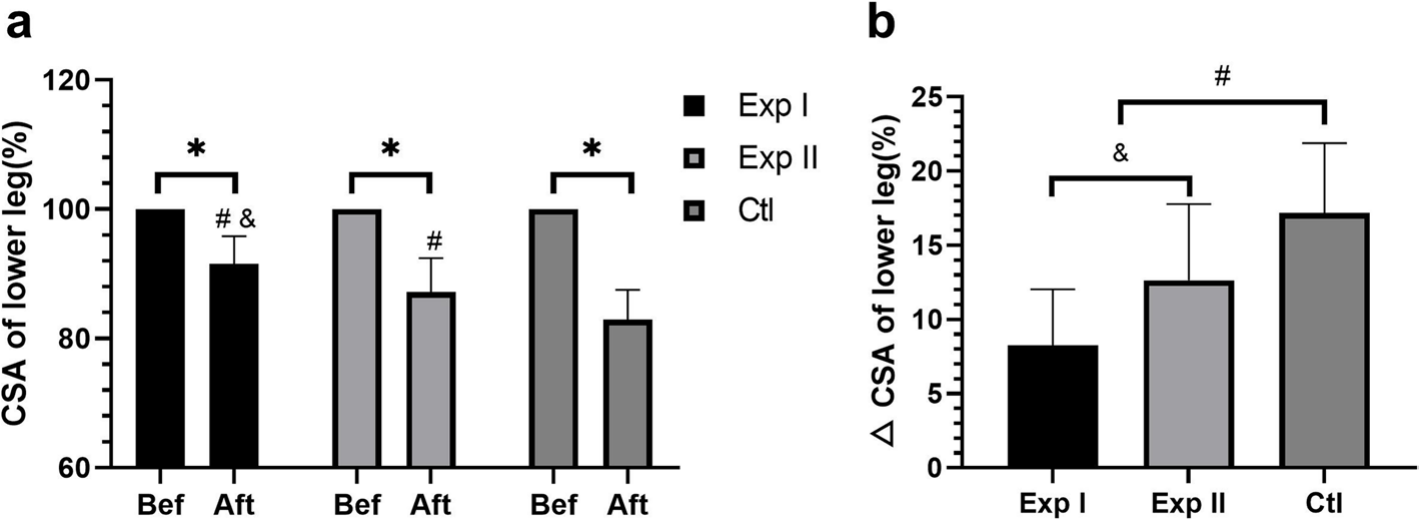
Comparison of muscle CSA of the lower leg before and after treatment. **a** Decrease of muscle CSA of the lower leg before and after treatment among three groups. **b** Comparison of muscle CSA gap before and after treatment in three groups. Exp I: experimental group I, Exp II: experimental group II, Ctl: control group; Bef: before treatment, Aft: after treatment; CSA: cross-sectional area; * *P* < 0.05 between each group before and after treatment; ^#^ *P* < 0.05 vs. Ctl; ^&^ *P* < 0.05 between Exp I and the Exp II

Compared with before treatment, muscle CSA in three groups decreased in different ranges (all *P* < 0.05). The comparative analysis between groups showed that after treatment, the decrease of muscle CSA in the leg of Exp I and Exp II was smaller than that of the Clt (Exp I vs Ctl, d = 8.93, 95%CI:6.20 ~ 11.66, *P* < 0.01; Exp II vs Ctl, d = 4.54, 95%CI: 1.38 ~ 7.70, *P* < 0.05), and there was a significant difference between Exp I and Exp II (d = 4.39, 95%CI: 1.50 ~ 7.28, *P* < 0.05) (Fig. 7).

### Secondary outcome - safety parameters

**Fig. 8:**
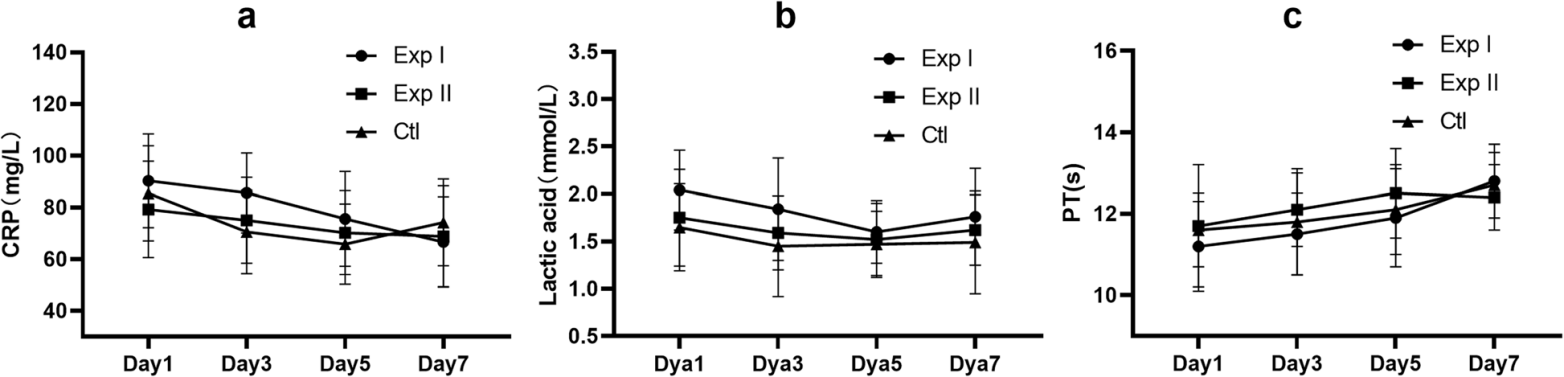
Comparison of the changes of CRP (**a**), lactic acid (**b**), and PT (**c**) in the three groups during treatment. Exp I: experimental group I, Exp II: experimental group II, Ctl: control group

There was no statistically significant difference in CRP, lactic acid, and PT among the three groups on the 1st, 3rd, 5th^,^ and 7th days of treatment (Fig. 8).

## Discussion

Using NMES to prevent muscle atrophy in ICU patients is still controversial [11, 16–19]. Our results showed that even with exercise therapy and NMES treatment, patients without nerve injury in ICU still developed muscle atrophy. Yet, stimulating antagonistic muscles simultaneously with NMES provided a better curative effect that when stimulating a group of muscles alone.

Myogenic weakness occurs in ICU patients due to systemic inflammatory response, stress, heavy drug use, and reduced stress load [26]. Comprehensive treatment is generally adopted, including nutritional support, blood sugar control, and rehabilitation treatment [27–29]. During the rehabilitation treatment, active and passive exercise training for patients is generally considered. Most scholars [30] have approved NMES treatment for patients with nerve injury or inability to move; yet, it is still controversial whether NMES should be added to patients with no nerve injury, consciousness, and active bed exercise. Some scholars advocate that when the level of consciousness of patients is improved, NMES is no longer needed when voluntary activities can be carried out [13]; however, there is still a lack of evidence-based medical support for whether NMES can increase the therapeutic effect of active and passive movement.

This study showed that despite the early APAT intervention, the patients still had a significant decline in muscle strength, while the patients who received NMES intervention in combination with APAT had a relatively small decline in muscle strength (*P* < 0.05), which is consistent with results reported by Akar et al. [31] It is believed that NMES may promote muscle activity that cannot be stimulated by exercise. We suspect that the possible mechanism is that the recruitment of motor units in NMES is just the opposite of active muscle contraction and that larger myocytes with lower axonal input impedance are more likely to be excited, and large motor units are more likely to be recruited [32]. It appears that electrically evoked muscle action produces more force than active contraction [33]. Therefore, for awake patients in ICU, early APAT combined with NMES can have a greater effect on muscular atrophy.

Some studies have reported contrary views on using NMES to prevent muscular atrophy. Some recommended stimulating quadriceps femoris only [34, 35], while others suggested stimulating multiple muscles [13] or agonist/antagonist muscle alone [36]. The present study showed that NMES single muscle group stimulation promoted slow ankle ROM loss (which is consistent with Shamsi et al [37]) and lowered extremity muscle atrophy (which is consistent with Dirks et al [38]); still, this effect was higher when a combination therapy (NMES agonist/antagonist muscle) was used. A possible reason for this result is that the simultaneous stimulation of the agonist/antagonist muscle can generate and transmit the resistance across the ankle joint through the tendon, forming the centrifugal activity of the muscle, which is the protective element of joint stability or resistance to articular cartilage stress [39]. Westing et al [40] found that the muscle torque produced by electrically stimulated eccentric contraction was 21 to 24% greater than that produced by eccentric autonomic contraction. Other studies have shown that eccentric contraction may slow the onset of muscle fatigue by increasing muscle torque [41]. In addition, other studies have also found that the co-contraction of the agonist/antagonist muscle may produce higher loads, and the co-activation of the agonist/antagonist muscle can significantly increase the tension of the Achilles tendon that can further effectively stretch the ankle joint [42, 43]. Therefore, the simultaneous stimulation of the agonist/antagonist muscle can improve the joint’s ROM. However, the specific mechanism needs to be verified by further experimental studies.

When NMES is used in ICU patients, its safety and possible side effects should also be considered. Common side effects are skin burns, muscle soreness, increased lactic acid, etc. Most of the side effects are caused by improper setting of treatment parameters or poor skin and muscle function of patients. Yet, in this study, no obvious skin burns and muscle soreness were observed. In addition, the rapid changes in inflammatory factors and coagulation function in patients after trauma can reflect the progress of the disease [44, 45]. Therefore, this study monitored the changes in CRP and PT in patients during NMES treatment; the results showed that NMES intervention had no significant effect on patients’ inflammatory status and coagulation function. Therefore, NMES is considered relatively safe for severe post-traumatic patients.

This study has a few limitations. Firstly, the sample size is relatively small. Secondly, because the purpose of this study is to evaluate the efficacy of early NMES intervention, the duration of NMES intervention is relatively short. Thus, our data need to be further verified in large sample size studies with a longer follow-up. In addition, most of the patients in this study are severe trauma patients; therefore, the findings may not apply to patients with ICU-AW due to severe cardiopulmonary and neurologic diseases.

## Conclusion

Although the overall trend of muscle atrophy cannot be reversed in ICU patients without nerve injury, the combination of exercise and NMES can significantly slow down its development. In addition to APAT, NMES should be used to prevent muscle atrophy in the early stage, and the agonist/antagonist muscle should be simultaneously stimulated to enhance the effect of preventing muscle atrophy. It is relatively safe to perform NMES in the early phase of recovery.

## Data Availability

The datasets generated and analyzed during the current study are not publicly available due to the privacy concern of participants but are available from the corresponding author upon reasonable request.

## Abbreviations

NMES: Neuromuscular Electrical Stimulation
ICU: Intensive Care Unit
Exp I: Experimental Group I
Exp II: Experimental Group II
Ctl: Control Group
APAT: Active and passive activity training
ICU-AW: Intensive care unit-acquired weakness
CSA: Cross-sectional area
AROM: Active range of motion
CRP: C-reactive protein
PT: Prothrombin time
PPS: Per Protocol Set
